# Review of the safety and efficacy of vitamin A supplementation in the treatment of children with severe acute malnutrition

**DOI:** 10.1186/1475-2891-12-125

**Published:** 2013-09-12

**Authors:** Lora L Iannotti, Indi Trehan, Mark J Manary

**Affiliations:** 1 Institute for Public Health/George Warren Brown School of Social Work, Washington University in St. Louis, Campus Box 1196, St. Louis, MO 63130, USA; 2 Department of Pediatrics, Washington University in St. Louis, 1 Childrens Pl, St Louis, MO 63110, USA; 3 Department of Paediatrics and Child Health, University of Malawi, Blantyre, Malawi; 4 Department of Community Health, University of Malawi, Blantyre, Malawi; 5 Children’s Nutrition Research Center, Baylor College of Medicine, Houston, Texas, USA

**Keywords:** GRADE, Vitamin A supplementation, Severe acute malnutrition, Systematic review

## Abstract

**Background:**

World Health Organization (WHO) guidelines recommend for children with severe acute malnutrition (SAM), high-dose vitamin A (VA) supplements be given on day 1 of admission, and on days 2 and 14 in the case of clinical signs of vitamin A deficiency (VAD). Daily low-dose VA follows, delivered in a premix added to F-75 and F-100. This study aimed to systematically review the evidence for safety and effectiveness of high-dose VA supplementation (VAS) in treatment of children with SAM.

**Methods:**

A comprehensive literature review was undertaken for all relevant randomized controlled trials (RCT) and observational studies from 1950 to 2012. Studies identified for full review were evaluated using the Grading of Recommendations, Assessment, Development and Evaluation (GRADE) methodology using a set of pre-defined criteria: indirectness; inconsistency; imprecision; and study limitations. A quality rating of *high, moderate,* or *low* was then assigned to each study, and only those attaining *moderate* to *high* were considered in making recommendations.

**Results:**

Of the 2072 abstracts screened, 38 met criteria for full review, and 20 were rated *moderate* to *high* quality. Only one study replicated the WHO VA protocol in children with SAM. *Indirectness* was a critical limitation, as studies were not exclusive to children with SAM. There was *inconsistency* across trials for definitions of malnutrition, morbidities, and ages studied; and *imprecision* arising from sub-group analyses and small sample sizes. Evidence showed improved outcomes associated with low-dose compared to high-dose VAS, except in cases presenting with signs of VAD, measles, and severe diarrhea or shigellosis. Adverse outcomes related to respiratory infection, diarrhea, and growth were associated with high-dose VAS in children who were predominantly adequately nourished. No adverse effects of the high dose were found in children with SAM in the trial that replicated the WHO VA guideline.

**Conclusion:**

This is the first systematic review of the safety and efficacy of high-dose VAS in treatment of SAM. We recommend a low-dose VAS regimen for children with SAM, except in cases presenting with measles, severe diarrhea (shigellosis), and any indication of VAD. Further research is needed in exclusively malnourished children and to explore alternate delivery strategies.

## Background

Globally, vitamin A deficiency (VAD) affects 100–140 million children, 4.4 million of whom have xerophthalmia [1,2]. Coverage rates for full vitamin A supplementation (VAS) of children 6–59 months delivered through primary care have risen significantly over the last decade, reaching 88% for the least developed countries [3]. In recent years, alternative strategies to improve micronutrient nutrition have been increasingly applied for high-risk populations already supplemented with vitamin A (VA): micronutrient powders; fortification of staple foods; ready-to-use supplemental and therapeutic foods (RUSF and RUTF); and other food-based approaches.

Among the populations particularly vulnerable to VAD are children with severe acute malnutrition (SAM) [4]. The World Health Organization (WHO) currently recommends that for inpatient care of children with SAM, VA supplements be given on day 1 of admission unless there is clear evidence that VA was received in the last month [5]. Dosing guidelines are the following: 200,000 international units (IU) for children over 12 months of age, 100,000 IU for children 6–12 months, and 50,000 IU for children below 6 months. If clinical signs of vitamin A deficiency are present, then another age-specific, large dose is administered on day 2 and again, on day 14 [4,5]. Low-dose VA is then given as part of a vitamin mix added to F-75 or F-100 therapeutic milk formulations, or alternatively, as a proprietary multivitamin supplement or combined mineral-vitamin mix (CMV). There is a need to revisit these guidelines in the context of improved VA availability, but also in view of evidence showing the potential for harmful effects of such VAS dosages [6].

## Methods

This systematic review aimed to assess the safety and effectiveness of VAS in children with SAM, with regards to mortality, nutritional recovery, and signs of symptomatic VAD [7]. A literature review was undertaken to search for all randomized controlled trials (RCT) and observational studies published from 1950 to March 2012. Databases searched included MEDLINE, EMBASE, and Google Scholar. The clinical trial registries at clinicaltrials.gov, pactr.org, and apps.who.int/trialsearch were also searched. Initial key words for the searches included “malnutrition”, “severe malnutrition”, “kwashiorkor”, “marasmus”, “vitamin A”, and “retinol”. A number of outcome measures were sought, including mortality, weight gain, nutritional recovery, and VAD. Further terms were added iteratively to the search based on results obtained from the initial searches.

Searches were also conducted to identify relevant publications and study documents produced by international health organizations such as the WHO and UNICEF. The titles and abstracts were scanned to identify relevant studies. The full text of relevant studies was obtained and the list of articles for inclusion further optimized based on an evaluation of the full text. Reference lists in relevant articles were also scanned manually and electronically to identify other studies that may have been missed in the original searches. Relevant publications that cited those previously identified articles were similarly sought.

Studies were included for full review based on relevance to the population of interest, study interventions, study design, outcome measures, and an assessment of the study’s methodological rigor and quality. These studies were then assessed and entered into a Grading of Recommendations, Assessment, Development and Evaluation (GRADE) table summarizing design, aim, outcomes, GRADE criteria assessment (indirectness, inconsistency, imprecision, and study limitations), and quality ranking (high, moderate, or low) [8].

*Indirectness* was judged based on study relevance to the review question in terms of the study population (children with SAM), intervention of interest (VAS), and outcome measures (mortality, recovery from SAM, signs of symptomatic VAD, and adverse outcomes due to supplementation) [9]. Consideration was given under this category to VA dosing relative the WHO protocol. *Inconsistency* was assessed by comparing point estimates and examining the heterogeneity of methods and statistical analyses [10]. *Imprecision* was based primarily on the 95% confidence interval with consideration of effect and sample sizes [11]. Potential biases (selection, recall, information/observation, misclassification) arising from failure to blind, losses in follow-up, inappropriate controls, and failure to adjust for confounding factors, among other problems, were considered under the *study limitations (risk of bias)* criteria [12].

Once these criteria were assessed, each study was assigned a quality ranking ranging from low to high. Only those studies with some degree of moderate or high (*i.e.*, low-moderate, moderate, moderate-high, or high) quality ranking were included in this review for consideration in making recommendations on the use of VAS for the treatment of children with SAM.

## Results

**Figure 1:**
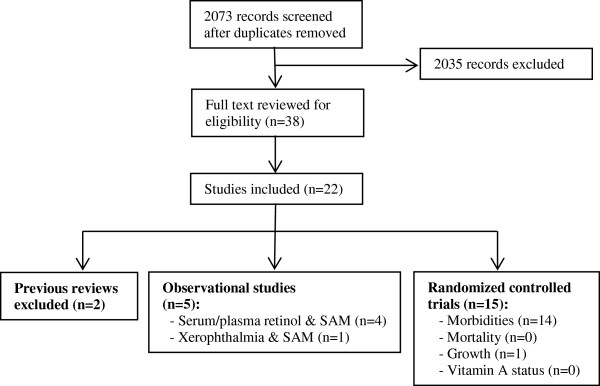
**Flow diagram for studies included in review.** The figure presents a flow diagram detailing the number of records screened and excluded, full-text articles reviewed, and ultimately, the number of studies included in the systematic review. The included observational and RCT studies are enumerated according to the primary outcome examined.

Of the 2072 abstracts identified and screened, 38 were selected for full review (Figure 1). Twenty-two were of moderate- to high-quality, and were grouped into 3 categories and listed primary outcomes assessed: 1) previous reviews; 2) observational studies; and 3) randomized controlled trials (RCT).

### Previous reviews

Two previous reviews of VAS trials were identified; both considered malnourished children together with those not malnourished [7,13]. Preventive VAS reduced all-cause mortality by 25% and diarrhea-specific mortality by 30%. VA showed greater protection against morbidity and mortality in Asia than in Africa or Latin America [13]. An earlier review found that the severity of measles and diarrheal infections were reduced by VAS, but the risk of lower respiratory tract infection was increased in some trials [7]. Because the reviews did not provide new evidence for VA and management of SAM and all studies from the reviews were screened for this review, they were not included in the GRADE assessment.

### Observational studies

Five observational studies were identified and assigned a GRADE quality ranking of low to moderate [14-18] (Additional file 1: Table S1). These studies found significant associations between serum or plasma retinol concentrations and SAM [14-16]. Others established a link between VAD, SAM, and diarrhea. In Bangladesh, reduced serum retinol concentrations were associated with shigellosis and low weight-for-age Z-score [18]. Another case–control study in Bangladesh showed that the duration of diarrhea and SAM were independently associated with xerophthalmia [17].

### Randomized controlled trials

Fifteen RCTs of moderate to high quality based on GRADE criteria were included in the systematic review on the safety and efficacy of high-dose VAS in children with SAM (Table 1). Issues of *indirectness* were present in all trials. Only one trial included children with SAM exclusively [19]. The remaining 14 included non-malnourished and malnourished children [20-33]. VA dosing also varied; only five trials administered the recommended high-dose on day 1 [19,26,30,31,33], while the remaining administered VA doses in varying quantities and forms. Only one trial followed the WHO protocol [19]; two compared high dose with daily low dose [26,33], and the remaining 12 compared high dose VAS with placebo [20-25,27-32].

**Table 1:** Randomized controlled trials included in the systematic review

**Quality Assessment**						**Summary of Findings**				**GRADE Rating**
						**Treatment Groups** (sample size)		**Effect**		
**Ref**	**Design**	**Indirectness**	**Inconsistency**	**Imprecision**	**Study Limitations** (risk of bias)	**Intervention Cases**	**Control Comparison**	**Relative Risk** (95% CI)	**Absolute**	
Sattar, 2012 [19]	RCT, double-blind	Pop: children 6-59 mo with SAM and diarrhea and/or ALRI	SAM def: WHZ < -3 or bipedal edema	No serious imprecision	No differences in baseline characteristics	High-dose and daily low-dose VA (n = 130)	Daily low-dose VA (n = 130)			MOD-HIGH
	Aim: test the efficacy and safety of WHO protocol for VAS in treatment of SAM among children 6-59 mo	VA dose: day 1: high-dose group, 200,000 IU (> 12 mo) or 100,000 IU (< 12 mo); low-dose group, placebo; day 2-15, 5000 IU daily for 15 days (both groups)			External validity: all children had diarrhea and low mean baseline serum retinol 13.15 ± 9.28 ug/dl	Resolution by 48 hr of acute diarrhea, dysentery, and ALRI			NS	
		Secondary outcome examined and found no increased risk of adverse events VA toxicity and morbidities.				Duration of acute diarrhea, invasive diarrhea, cough, fever, rales, edema, and skin changes			NS	
						Changes in nutritional status, weight, length, MUAC, head circumference			NS	
						Incidence of nosocomial diarrhea		aOR 1.25 (0.67, 2.34)	NS	
						and ALRI infections		aOR 0.63 (0.36-1.09)	NS	
Donnen, 2007 [33]	RCT, double-blind	Pop: 44-48% WHZ < -2; 30-37% MUAC < 125 mm	SAM def: WHZ < -2; MUAC < 125 mm	No serious imprecision	Age confounding: high-dose group younger	Daily low-dose VA (n = 610)	Single high-dose VA and placebo daily (n = 604)			MOD
	Aim: assess effect of single high-dose vs. daily low-dose VA supplements on hospitalized malnourished Senegalese children’s (0 - 14 yr) morbidity	VA dose: high-dose: single 200,000 IU (> 12 mo) or 100,000 IU (< 12 mo); low-dose: 5000 IU daily until discharge			Baseline nutrition: MUAC lower in high-dose group	Incidence of respiratory disease		HR 0.26 (0.07 - 0.92)	p < 0.05	
		Sub-group: children with edema mortality lower in low-dose (AOR 0.21, 0.05-0.99)				Duration: respiratory disease		HR of cure 1.41 (1.05 - 1.89)	p = 0.019	
						Incidence & duration of diarrhea		HR 1.02 (0.68 - 1.52)	NS	
Mahalan-abis, 2004 [32]	RCT, factorial	Pop: children with marasmus or edema excluded	No serious inconsistency	Small sample	Baseline nutrition: serum retinol was 0.387 μmol/L higher (P = 0.001) in VA treated group	zinc + VA (n = 38) zinc (n = 39) VA (n = 38)	Placebo (n = 38)			LOW-MOD
	Aim: evaluate effect of zinc and VA on clinical recovery of Indian children (2 - 24 mo) with severe acute lower respiratory infections	VA dose: 10,000 μg RE 2x/d for 4d; zinc 10 mg 2x/d for 5d				Recovery rates from very ill status zinc (boys)		2.63 (1.35 - 5.10)		
						Recovery rate from fever zinc		3.12 (1.47 - 6.6)	NS	
						Recovery rates for VA				
						**Adverse event risk** increased risk of diarrhea in VA group			p = 0.028	
						increased risk of any adverse events in VA group		RR 3.8 (1.32-10.93)		
Fawzi, 2000 [31]	RCT	VA dose: given on days 0 and 2, and again at 4 and 8 mo; dose after discharge, 200,000 IU (> 1 yr) or 100,000 IU (infants)	SAM def: NCHS references; WHZ < -2	Statistics: few variables studied in multivariate analyses	Potential seasonal bias: enrollment during 1993-1997	VA (n = 289)	Placebo (n = 285)			LOW-MOD
	Aim: determine effect of VAS on risk of diarrhea and acute respiratory infections in hospitalized Tanzanian children (6 - 60 mo)	Sub-group: **adverse event risk** normally nourished children: VA increased risk of acute diarrhea; wasted children protective against diarrhea	Diarrhea def: mother’s perception; 3+ days elapsed between episode; persistent diarrhea defined as lasting 14+ days			Severe watery diarrhea		AOR 0.56 (0.32 - 0.99)		
						Hospitalization			NS	
						**Adverse event risk** cough and rapid respiratory rate		AOR 1.67 (1.17 - 2.36)		
Faruque, 1999 [29]	RCT, factorial	Pop: 34-35% WAZ < -2; acute diarrhea ≤ 3 days	Diarrhea def: mother’s perception with picture	No serious imprecision	No differences observed by stunting at baseline	Group A: VA 15d (n = 170) Group B: zinc (n = 172) Group C: VA + zinc (n = 171)	Placebo (n = 171)			MOD
	Aim: to assess efficacy of zinc or VAS in Bangladeshi children (6 mo - 2 yr) with acute diarrhea	VA dose: 4500 μg RE for 15d	Children observed in hospital 24 h and followed at home 15d			Zinc groups Diarrhea duration reduced by 13 %			p = 0.03	
		Zinc dose: 14.2 - 40 mg zinc for 15d				Prolonged diarrhea reduced by 43%			p = 0.017	
						VA groups Prolonged diarrhea			NS	
						Overall diarrhea duration			NS	
Donnen, AJCN 1998 [26]	RCT, double-blind	Pop: 26-28% WHZ < -2; 20-29% MUAC < 125 mm	SAM def: WHZ < -2, MUAC < 125 mm	Small effect size	Baseline CRP elevated in high dose group	Daily low-dose VA (n = 298)	High-dose VA (n = 300)			MOD
	Aim: assess effect of single high-dose vs. daily low-dose VA supplements on hospitalized pre-school aged children in Democratic Republic of Congo	VA dose: high-dose: single 200,000 IU (> 12 mo) or 100,000 IU (< 12 mo) at admission; low-dose: 5000 IU daily until discharge				Sub-group: SAM (low dose VA vs. placebo) Incidence of severe diarrhea in SAM	Placebo (n = 302)	RR 0.21(0.07 - 0.62)		
		**Adverse event risk** Sub-group: children with no edema increased risk of diarrhea with high-dose VA compared to placebo (AOR 2.42, 1.15 - 5.11)				Duration of: severe diarrhea			NS	
						Acute lower respiratory infection			NS	
						Fever			NS	
Donnen, J of Nutr. 1998 [20]	RCT	VA dose: 60 mg oily solution of retinyl palmitate (30 mg for children < 12 mo)	SAM def: WHZ < -2, MUAC < 125 mm; NCHS standards; discharged children		Failure to blind: no placebo used	Group 1: VA baseline and at 6 mo (n = 118)	Group 3: placebo (n = 117)			MOD
	Aim: assess effect of single high-dose VA supplements and regular antiparasitic therapy on growth of moderately malnourished children (0 - 72 mo) from eastern Zaire	Pop: 4-9% WHZ < -2			Unclear why changes in Z-scores not compared	Group 2: Mebendazole every 3 mo for 1 yr (n = 123)				
		Sub-group: VA boys gain more weight and height				Group 1: Annual weight gain 2.09 kg	Group 3: Annual weight gain 1.18 kg		p = 0.029	
						MUAC gain 2.24 cm	MUAC gain 0.95 cm		p = 0.012	
						Group 2 vs. 3 Growth			NS	
						**Adverse risk:** VA girls gained less height than controls. De-wormed boys and girls gained less weight than control boys and girls				
Stephen-sen, 1998 [28]	RCT, double-blinded	VA dose: children ≤ 1 yr: 100,000 IU on day 1 and 50,000 IU on day 2; children > 1 yr: 200,000 IU on day 1 and 100,000 IU on day 2	SAM def: US reference population	Small sample	Potential confounding: WHZ lower in VA group at baseline	VA (n = 48)	Placebo (n = 49)			LOW-MOD
	Aim: test hypothesis that high-dose VA supplements will enhance recovery of Peruvian children (3 mo - 10 yrs) hospitalized with pneumonia			Statistics: univariate comparisons reported primarily without adjusting for covariates	Selective reporting	**Adverse events:** ↓ blood oxygen saturation	**Adverse events:** ↑blood oxygen saturation		p < 0.05	
						Prevalence of retractions 37%	Prevalence of retractions 15%			
						Auscultatory evidence of consolidation 28%	Auscultatory evidence of consolidation 17%			
						supplemental oxygen need 21%	supplemental oxygen need 8%			
						Duration of hospitalization			NS	
						Chest x-rays			NS	
Nacul, 1997 [24]	RCT, double-blind placebo	VA dose: 200,000 IU (< 12 mo); 400 000 IU (> 12 mo)	Pop: some hospitalized while others outpatient	No serious imprecision	Low serum retinol at baseline in both groups likely due to infection; serum retinol in VA group higher on day 11	VA (n = 239)	Placebo (n = 233)			MOD-HIGH
	Aim: evaluate impact of VAS on clinical recovery and severity of pneumonia among Brazilian children (6 - 59 mo)	Subgroup analysis showed beneficial effect on those most severely affected with VA deficiency				Fever by day 3: 16 %	Fever by day 3: 26.4 %		p = 0.008	
					Transitory bulging fontanelle (4%)	Failure to respond to first line antibiotic		rate ratio 0.71 (0.50 - 1.01)	p = 0.054	
					No effect attributable to etiological agent	Duration of pneumonia			NS	
						Incidence of adverse outcomes			NS	
Julien, 1999 [30]	RCT, double-blind placebo	VA dose: 200,000 IU (>12 mo); 100,000 IU (< 12 mo)		Small sample size to detect morbidity differences	VA deficiency high at baseline in both groups: 68.9% had serum retinol <10 μg/dL and 93.2% serum retinol < 20 μg/dL	VA (n = 71)	Placebo (n = 93)			LOW-MOD
	Aim: test the potential of VAS at admission to speed up recovery during hospitalization for lower respiratory infection and decrease morbidities at 6 wk in Mozambican children (6 - 72 mo)	Pop: kwashiorkor and marasmus excluded				Rate of clinical discharge on day 5: 88.4%	Rate of clinical discharge on day 5: 73.9%	RR 1.9 (1.01 - 3.05)	NS	
					No adjustment with covariates	6 wk Health care use Illness (fever, cough, or diarrhea)			NS	
Hossain, 1998 [27]	RCT, double-blind	Pop: excluded if WAZ ≤75% NCHS	Diarrhea def: liquid stools that can be poured or contain blood or mucus; clinical cure: ≤3 formed stools without visible blood or mucus	Small sample size	Baseline VA status not determined, but population prevalence high	VA (n = 42)	Control (n = 41)			LOW-MOD
	Aim: evaluate efficacy of single oral dose VA in treating shigellosis among Bangladeshi children (1 - 7 yr)				No adjustment with covariates	Clinical cure 19/24 (45%)	Clinical cure 8/14 (20%)	risk ratio 0.68 (0.50 - 0.93)	p = 0.02	
							Bacteriological cure 16/42 (38%)			
		Bacteriological cure 16/41 (39%)	risk ratio 0.98 (0.70 - 1.39)	NS						
Si, 1997 [25]	RCT, double-blind	Pop: SAM excluded; 45% moderately underweight	Nutrition def: moderate malnutrition defined as WAZ 60-80% of NCHS median	No confidence intervals provided	Potential selection bias: trend for greater pneumonia severity in VA group (p = 0.06)	VA (n = 280)	Placebo (n = 312)		NS	LOW-MOD
	Aim: evaluate effect of high dose VA supplement on morbidity in hospitalized Vietnamese children (1 - 59 mo) with pneumonia	VA dose: 200,000 in peanut oil (< 1 yr); 400,000 IU in peanut oil (1 - 4 yr)			No baseline VA status	Mean time to normal: fever respiratory rate			NS	
		Sub-group analysis: moderately malnourished girls showed shorter duration of hospitalization			No adjustment for covariates	Duration of hospitalization			NS	
Dibley, 1995 [23]	RCT, double-blind	Pop: 3.4% wasted; 37% stunted	Morbidity def: 3+ loose stools per day; time between episodes 2+ diarrhea-free days	Small effect size for acute respiratory infection; large CI for acute lower respiratory infection	Baseline difference in immunization status: ever vaccinated for polio and measles higher in VA group	VA (n = 396)	Placebo (n = 386)			MOD-HIGH
	Aim: test efficacy of high-dose VA supplement on acute respiratory and diarrheal illnesses in Indonesian children (6 - 47 mo)	VA dose: 103,000 IU (< 1 yr); 206,000 IU (≥ 1 yr)	Acute respiratory episode with 2+ adjoining days for cough; time between episodes of 3+ symptom-free days			**Adverse event risks** Acute respiratory infection		rate ratio 1.08 (1.01 - 1.19)		
		**Adverse event risk:** Subgroup analysis showed VA increased diarrhea incidence in children < 30 mo				Acute lower respiratory infection		rate ratio 1.39 (1.00 - 1.93)		
						Diarrhea			NS	
Coutsou-dis, 1991 [22]	RCT, double blind	VA dose: 54.5 mg (< 12 mo); 109 mg (>12 mo)	Nut def: WAZ by NCHS	Small sample size	Losses to follow-up were 20% at 6 weeks and 40% at 6 months; no characteristics of this group provided	VA (n = 24)	Placebo (n = 24)			MOD
	Aim: test efficacy of VAS on measles morbidity in South African children (4 - 24 mo)		Morbidity def: diarrhea: 3+ loose or watery stools per day; pneumonia: presence of tachypnea with retractions, crackles, wheezes	Statistics: β set to 0.5 only to detect 30% increase in absolute recovery	No adjustment for covariates; only univariate analyses	IMS score 0.24 ± 0.15	IMS score 1.37 ± 0.40		p = 0.037	
			Integrated morbidity score (IMS) used			Weight gain at 6 wk 1.29 ± 0.17 kg	Weight gain at 6 wk 0.90 ± 0.14 kg		P = 0.04	
						Pneumonia recovery 3.8 ± 0.40 d	Pneumonia recovery 5.7 ± 0.79 d		P < 0.05	
Hussey, 1990 [21]	RCT, double-blind	Pop: 70/189 (37%) with WHZ < 5^th^%	Nutrition def: percentiles of NCHS standards	Rare events: mortality; pneumonia / diarrhea duration ≥10 days	Differences in baseline characteristics except rash duration and lower levels of total protein and albumin in placebo	VA (n = 92)	Placebo (n = 97)			MOD-HIGH
	Aim: test efficacy of high dose VAS on measles complications among hospitalized South African children	VA dose: 400,000 IU at admission			High prevalence of “hyporetinemia” (< 7.0 μmol /L) 76%	Pneumonia recovery 6.3 d	Pneumonia recovery 12.4 d		P < 0.001	
					No adjustment for covariates	Diarrhea recovery 5.6 d	Diarrhea recovery 8.5 d		P < 0.001	
						Croup 13 cases	Croup 27 cases	0.51 (0.28 - 0.92)	P = 0.01	
						Days in hospital 10.6 d	Days in hospital 14.8 d	0.21 (0.05 - 0.94)		
						Mortality 2 deaths	Mortality 10 deaths			
						Risk of death due to major complication		RR 0.51 (0.35, 0.74)		

The table presents the included RCT studies in the review by date of publication, from most recent to oldest. For each study, the design, aim, quality assessment by GRADE criteria, findings, and GRADE rating are provided.

*Inconsistency* across trials was observed with regards to the ages of children studied, ranging from birth to 14 years. Definitions of morbidity and malnutrition varied, as well as use of differing growth references and standards. Small sample sizes and rare events were the most common problems related to *imprecision*. Despite randomization, several studies also showed significant differences in baseline characteristics that elevated the *risk of bias*. Notably, these included baseline age and nutritional status differences with higher likelihoods of confounding [28,32,33].

Some key findings were identified across trials. First, low-dose VAS conferred more or similar health and recovery advantages when compared to high-dose supplementation in the treatment of malnourished children [19,26,33]. Second, high-dose VAS showed mixed results relative to placebo for infectious disease outcomes in children with and without SAM, showing benefit in some trials for children with severe diarrhea or shigellosis [27,31], measles [21,22], other acute respiratory infections [30], and undifferentiated fever [24]. However, in other trials, there was no benefit for children with acute respiratory infections or diarrhea [24-26,29,32]. There was also some evidence of improved growth outcomes associated with high-dose VAS among VA deficient children [20] and children with measles [22].

Finally, adverse effects were found to be associated with high-dose VAS in some trials, including an increased rate of respiratory illnesses [23,28] and diarrhea [26,31]. These findings appear primarily in samples or sub-samples of adequately nourished children. As noted previously, we identified only one RCT that included only severely malnourished children [19]. Some explicitly excluded children with marasmus, kwashiorkor, or all children with SAM [25,27,30,32], while the remaining included both malnourished and adequately nourished children. Among the five studies finding adverse events in association with VAS, two of these studies carried out sub-group analyses showing an increased risk of diarrhea among normally nourished children [31] and among children without edema [26]. One trial included only adequately nourished children [28], another found increased risk of ALRI in mainly adequately nourished children [23], and one included both malnourished and adequately nourished children, excluding children with SAM [32]. No adverse effects of the high-dose VAS were found in children with SAM in the trial that replicated the WHO VA guidelines [19].

## Discussion

Our systematic review of the literature using the GRADE methodology revealed only limited evidence that directly addresses the safety and effectiveness of high-dose VAS for children with SAM. Fourteen of the 15 RCTs identified for this review included both malnourished and non-malnourished children, thereby complicating the extrapolation of findings directly to efficacy and safety of VAS for SAM. There is sufficient evidence to recommend the use of high-dose VAS for children with SAM when presenting with measles, severe diarrhea (shigellosis), or evidence of VAD. The low-dose VAS regimen should be considered as the preferred protocol in other cases of malnourished children, given the potential for adverse events and similar recovery outcomes when compared to high-dose VAS. Higher quality prospective studies are still needed to directly examine VAD in children with SAM, applying more contemporary definitions of morbidity and malnutrition, and powered sufficiently to detect the critical outcomes of interest.

Observational studies demonstrated an association between SAM and VAD, but metabolic disruptions in SAM might preclude accurate measures of true VA status. Blood biomarkers of retinol concentrations were primarily used to indicate VA status [14-16,18]. Protein-energy malnutrition has been associated with reduced hepatic synthesis of retinol binding protein (RBP) and transthyretin used to transport VA in the body [34,35]. Thus, VA stores in the liver may not be mobilized appropriately in malnourished children and therefore not be reflected in blood biomarkers. Mitra showed that VA status improved from baseline to hospital discharge, even without VAS [18]. That study also showed that while urinary losses of retinol were associated with low retinol concentrations in children with dysentery, other inflammatory markers such as body temperature and serum α-1-acid glycoprotein and C reactive protein concentrations were more highly and negatively correlated with vitamin A status.

There is some evidence that diarrhea could be mediating this relationship, as revealed by independent associations between diarrhea, low WAZ, and VA levels [17]. Shigellosis*,* in particular, increases the risk of VAD [18]. A study in Brazil showed a possible interaction between SAM and low serum retinol concentrations for higher diarrheal morbidities [15]. Evidence from RCTs also finds that high-dose VAS may be beneficial for treating severely malnourished children in cases of severe diarrhea [27,31]. Again, the mechanism by which VA may be operating to mitigate severe diarrhea in SAM has not been elucidated, but it may be either through the repair of the gut epithelium or immune pathways that restore balance between T-cell subsets [36].

Two studies showed unequivocally the benefit of lose-dose (5000 IU daily until discharge) compared to a single high-dose (200,000 IU for children over 12 months or 100,000 IU for children under 12 months on day 1 of hospitalization) VAS regimen [26,33]. The low-dose regimen was more strongly correlated with lower incidence and shorter duration of respiratory disease [33] and a lower incidence of severe diarrhea [26]. A third study in Bangladesh also examined the administration of a lose-dose protocol in relation to a high-dose on day 1 followed by daily low doses until hospital discharge. [19]. That study did not find any differences in the two groups on a range of morbidity and nutrition recovery outcomes and adverse events. All children at baseline had diarrhea and while clinical signs of VAD were not apparent, baseline measures of VA status did indicate VAD in the sample. With regards to other morbidities, high-dose VAS was effective in reducing measles-specific respiratory illness [21,22], but showed mixed results with regards to non-measles pneumonia and other acute lower respiratory tract infections in children with SAM [23-25,28-30,32,37,38].

Only one trial identified in this review specifically included children who were HIV + [31]. Sub-group analyses stratified by HIV status showed a trend towards a protective effect of high-dose VAS on cough and rapid respiratory rate for HIV-infected children [RR 0.54 (0.24-1.20), p = 0.07], while children without HIV infection were at increased risk for these conditions [RR 1.47 (1.16-1.86), p = 0.001]. Because children infected with HIV are highly vulnerable to SAM and VAD, more research is needed to understand the optimal protocols for this population [39].

The evidence for growth outcomes in the follow-up period from hospital discharge was limited and somewhat dated [20,22]. One study showed that only among children who were VA deficient at baseline were there greater improvements in weight and MUAC in those who received high-dose VAS; this was not evident in the non-VA deficient children and high-dose VAS even reduced height gain among non-VA deficient girls [20]. Standard therapeutic foods designed for growth recovery in SAM may be a safer source of VA in certain cases. The foods contain VA as retinyl acetate, similar to VA supplements, in the following concentrations (per 100 g): F-75, 900 μg; F-100, 800 μg; and RUTF, 800–1100 μg. Thus, for a child weighing 5 kg, this would translate into 1,119 μg/d VA for F-75, or 1,544 μg/d VA for F-100, and 1,680 μg/d VA for RUTF. These quantities are comparable to the low-dose regimen of 5000 IU or 1500 μg retinol and are well below the high-dose recommendation of 100,000 IU or 30,000 μg/d VA that would be given to children under 12 mo. While these foods have not specifically been assessed as alternatives to VAS, the safety and efficacy of these products in the treatment of SAM is well-accepted [40-42]. Particularly in cases when a child’s VA status is unknown or adequate, therapeutic foods could thus serve as the delivery mechanism for low-dose VAS, though more research is needed.

Adverse effects found to be associated with high-dose VAS warrant more research. The most frequent negative outcome was an increased risk of acute respiratory infections [23,28,31,33]. There was also evidence of an increased risk of acute, non-severe diarrhea, mediated in part by nutritional status [26,31,32]. It should be noted that the adverse effects arising from high-dose VAS were found in samples or sub-samples that included primarily adequately nourished children. Heterogeneity in baseline characteristics and study design complicates comparisons across the various studies, but these safety issues should not be ignored. Excessive intake of preformed VA, notably in the absence of dietary fatty acids, may overwhelm the esterification process and introduce more harmful forms of VA into the child’s circulation [6]. Further along the metabolic pathway, VA presented to cell membranes in forms other than the RBP complex (as may be the case in SAM) can also lead to significant cellular damage [43]. Carotenoid forms of VA or lipid-based nutrient supplements such as RUTF, should be explored to minimize oxidative stress while still addressing VAD [44].

## Conclusions

In the treatment of children with SAM, a high-dose VAS protocol can be safely recommended in cases presenting with measles, severe diarrhea (shigellosis), or symptoms of VAD. More research is needed to study this specific question in populations exclusively malnourished and to understand and prevent adverse outcomes related to high-dose VAS. We recommend exploration of alternative low-dose protocols and strategies beyond VAS, such as use of carotenoids or RUTF interventions, to address VA deficiency and associated health outcomes in the treatment of children with SAM.

## Abbreviations

ALRI: Acute lower respiratory infection; GRADE: Grading of recommendations assessment development and evaluation; MUAC: Mid-upper arm circumference; RCT: Randomized controlled trial; RUTF: Ready-to-use therapeutic food; SAM: Severe acute malnutrition; VA: Vitamin A; VAD: Vitamin A deficiency; VAS: Vitamin A supplementation; WAZ: Weight-for-age Z score; WHZ: Weight-for-height Z score.

## Competing interests

There were no competing interests in this research.

## Authors’ contributions

LLI, IT, and MJM contributed to the conception and design of the study. IT and MJM supervised the literature search and initial screening of studies. LLI carried out the full text review and initial GRADE assessment, followed by additional assessment from IT and MJM. All authors contributed to the analysis and interpretation of the compiled evidence. LLI drafted the manuscript with significant inputs received by IT and MJM. All authors have granted approval to this version of the manuscript.

## Supplementary Material

Additional file 1: Table S1 Observational studies included in systematic review [45]. Click here for file

## References

[B1] Black RE Allen LH Bhutta ZA Caulfield LE De Onis M Ezzati M Mathers C Rivera J Maternal and child undernutrition: global and regional exposures and health consequences Lancet 2008 371 243 260 10.1016/S0140-6736(07)61690-0 18207566

[B2] West KP Jr Extent of vitamin A deficiency among preschool children and women of reproductive age J Nutr 2002 132 2857S 2866S 12221262 10.1093/jn/132.9.2857S

[B3] UNICEF The State of the World's Children 2012 2012 New York: United Nations Children’s Fund

[B4] WHO Management of severe malnutrition: a manual for physicians and other senior health workers 1999 Geneva: World Health Organization

[B5] Ashworth A Khanum S Jackson A CS Guidelines for the inpatient treatment of severely malnourished children 2003 Geneva: World Health Organization

[B6] Penniston KL Tanumihardjo SA CS The acute and chronic toxic effects of vitamin A Am J Clin Nutr 2006 83 191 201 16469975 10.1093/ajcn/83.2.191

[B7] Villamor E Fawzi WW Vitamin A supplementation: implications for morbidity and mortality in children J Infect Dis 2000 182 S122 133 10.1086/315921 10944494

[B8] Atkins D Best D Briss PA Eccles M Falck-Ytter Y Flottorp S Guyatt GH Harbour RT Haugh MC Henry D Grading quality of evidence and strength of recommendations BMJ 2004 328 1490 15205295 10.1136/bmj.328.7454.1490 PMC428525

[B9] Guyatt GH Oxman AD Kunz R Woodcock J Brozek J Helfand M Alonso-Coello P Falck-Ytter Y Jaeschke R Vist G GRADE guidelines: 8. Rating the quality of evidence--indirectness J Clin Epidemiol 2011 64 1303 1310 10.1016/j.jclinepi.2011.04.014 21802903

[B10] Guyatt GH Oxman AD Kunz R Woodcock J Brozek J Helfand M Alonso-Coello P Glasziou P Jaeschke R Akl EA GRADE guidelines: 7. Rating the quality of evidence--inconsistency J Clin Epidemiol 2011 64 1294 1302 10.1016/j.jclinepi.2011.03.017 21803546

[B11] Guyatt GH Oxman AD Kunz R Brozek J Alonso-Coello P Rind D Devereaux PJ Montori VM Freyschuss B Vist G GRADE guidelines 6. Rating the quality of evidence--imprecision J Clin Epidemiol 2011 64 1283 1293 10.1016/j.jclinepi.2011.01.012 21839614

[B12] Guyatt GH Oxman AD Vist G Kunz R Brozek J Alonso-Coello P Montori V Akl EA Djulbegovic B Falck-Ytter Y GRADE guidelines: 4. Rating the quality of evidence--study limitations (risk of bias) J Clin Epidemiol 2011 64 407 415 10.1016/j.jclinepi.2010.07.017 21247734

[B13] Imdad A Yakoob MY Sudfeld C Haider BA Black RE Bhutta ZA Impact of vitamin A supplementation on infant and childhood mortality BMC Publ Health 2011 11 Suppl 3 S20 10.1186/1471-2458-11-S3-S20 PMC3231894 21501438

[B14] Ashour MN Salem SI El-Gadban HM Elwan NM Basu TK Antioxidant status in children with protein-energy malnutrition (PEM) living in Cairo, Egypt Eur J Clin Nutr 1999 53 669 673 10.1038/sj.ejcn.1600830 10477255

[B15] De Fatima Costa Caminha M Da Silva Diniz A Falbo AR De Arruda IK Serva VB De Albuquerque LL De Freitas Lola MM Ebrahim GJ Serum retinol concentrations in hospitalized severe protein-energy malnourished children J Trop Pediatr 2008 54 248 252 10.1093/tropej/fmn018 18385151

[B16] Donnen P Brasseur D Dramaix M Vertongen F Ngoy B Zihindula M Hennart P Vitamin A deficiency and protein-energy malnutrition in a sample of pre-school age children in the Kivu Province in Zaire Eur J Clin Nutr 1996 50 456 461 8862482

[B17] Mahalanabis D Breast feeding and vitamin A deficiency among children attending a diarrhoea treatment centre in Bangladesh: a case–control study BMJ 1991 303 493 496 10.1136/bmj.303.6801.493 1912858 PMC1670801

[B18] Mitra AK Alvarez JO Wahed MA Fuchs GJ Stephensen CB Predictors of serum retinol in children with shigellosis Am J Clin Nutr 1998 68 1088 1094 9808227 10.1093/ajcn/68.5.1088

[B19] Sattar S Ahmed T Rasul CH Saha D Salam MA Hossain MI Efficacy of a high-dose in addition to daily low-dose vitamin A in children suffering from severe acute malnutrition with other illnesses PloS ONE 2012 7 e33112 10.1371/journal.pone.0033112 22479361 PMC3314008

[B20] Donnen P Brasseur D Dramaix M Vertongen F Zihindula M Muhamiriza M Hennart P Vitamin A supplementation but not deworming improves growth of malnourished preschool children in eastern Zaire J Nutr 1998 128 1320 1327 9687551 10.1093/jn/128.8.1320

[B21] Hussey GD Klein M A randomized, controlled trial of vitamin A in children with severe measles N Engl J Med 1990 323 160 164 10.1056/NEJM199007193230304 2194128

[B22] Coutsoudis A Broughton M Coovadia HM Vitamin A supplementation reduces measles morbidity in young African children: a randomized, placebo-controlled, double-blind trial Am J Clin Nutr 1991 54 890 895 1951162 10.1093/ajcn/54.5.890

[B23] Dibley MJ Sadjimin T Kjolhede CL Moulton LH Vitamin A supplementation fails to reduce incidence of acute respiratory illness and diarrhea in preschool-age Indonesian children J Nutr 1996 126 434 442 8632216 10.1093/jn/126.2.434

[B24] Nacul LC Kirkwood BR Arthur P Morris SS Magalhaes M Fink MC Randomised, double blind, placebo controlled clinical trial of efficacy of vitamin A treatment in non-measles childhood pneumonia BMJ 1997 315 505 510 10.1136/bmj.315.7107.505 9329303 PMC2127384

[B25] Si NV Grytter C Vy NN Hue NB Pedersen FK High dose vitamin A supplementation in the course of pneumonia in Vietnamese children Acta Paediatr 1997 86 1052 1055 10.1111/j.1651-2227.1997.tb14805.x 9350882

[B26] Donnen P Dramaix M Brasseur D Bitwe R Vertongen F Hennart P Randomized placebo-controlled clinical trial of the effect of a single high dose or daily low doses of vitamin A on the morbidity of hospitalized, malnourished children Am J Clin Nutr 1998 68 1254 1260 9846855 10.1093/ajcn/68.6.1254

[B27] Hossain S Biswas R Kabir I Sarker S Dibley M Fuchs G Mahalanabis D Single dose vitamin A treatment in acute shigellosis in Bangladesh children: randomised double blind controlled trial BMJ 1998 316 422 426 10.1136/bmj.316.7129.422 9492664 PMC2665602

[B28] Stephensen CB Franchi LM Hernandez H Campos M Gilman RH Alvarez JO Adverse effects of high-dose vitamin A supplements in children hospitalized with pneumonia Pediatrics 1998 101 E3 9565436 10.1542/peds.101.5.e3

[B29] Faruque AS Mahalanabis D Haque SS Fuchs GJ Habte D Double-blind, randomized, controlled trial of zinc or vitamin A supplementation in young children with acute diarrhoea Acta Paediatr 1999 88 154 160 10.1111/j.1651-2227.1999.tb01074.x 10102147

[B30] Julien MR Gomes A Varandas L Rodrigues P Malveiro F Aguiar P Kolsteren P Stuyft P Hildebrand K Labadarios D Ferrinho P A randomized, double-blind, placebo-controlled clinical trial of vitamin A in Mozambican children hospitalized with nonmeasles acute lower respiratory tract infections Trop Med Int Health 1999 4 794 800 10.1046/j.1365-3156.1999.00493.x 10632986

[B31] Fawzi WW Mbise R Spiegelman D Fataki M Hertzmark E Ndossi G Vitamin A supplements and diarrheal and respiratory tract infections among children in Dar es Salaam, Tanzania J Pediatr 2000 137 660 667 10.1067/mpd.2000.110136 11060532

[B32] Mahalanabis D Lahiri M Paul D Gupta S Gupta A Wahed MA Khaled MA Randomized, double-blind, placebo-controlled clinical trial of the efficacy of treatment with zinc or vitamin A in infants and young children with severe acute lower respiratory infection Am J Clin Nutr 2004 79 430 436 14985218 10.1093/ajcn/79.3.430

[B33] Donnen P Sylla A Dramaix M Sall G Kuakuvi N Hennart P Effect of daily low dose of vitamin A compared with single high dose on morbidity and mortality of hospitalized mainly malnourished children in Senegal: a randomized controlled clinical trial Eur J Clin Nutr 2007 61 1393 1399 10.1038/sj.ejcn.1602671 17299466

[B34] Jain MK Mehta NJ Fonseca M Pai NV Correlation of serum vitamin A and its transport protein (RBP) in malnourished and vitamin A deficient children J Postgrad Med 1990 36 119 123 2102908

[B35] Rosales FJ Ritter SJ Zolfaghari R Smith JE Ross AC Effects of acute inflammation on plasma retinol, retinol-binding protein, and its mRNA in the liver and kidneys of vitamin A-sufficient rats J Lipid Res 1996 37 962 971 8725149

[B36] Semba RD Ward BJ Griffin DE Scott AL Natadisastra G West KP Jr Sommer A Muhilal Abnormal T-cell subset proportions in vitamin-A-deficient children Lancet 1993 341 5 8 10.1016/0140-6736(93)92478-C 8093291

[B37] Daulaire NM Starbuck ES Houston RM Church MS Stukel TA Pandey MR Childhood mortality after a high dose of vitamin A in a high risk population BMJ 1992 304 207 210 10.1136/bmj.304.6821.207 1739794 PMC1881470

[B38] Kjolhede CL Chew FJ Gadomski AM Marroquin DP Clinical trial of vitamin A as adjuvant treatment for lower respiratory tract infections J Pediatr 1995 126 807 812 10.1016/S0022-3476(95)70416-7 7752011

[B39] Trehan I O’Hare BA Phiri A Heikens GT Challenges in the Management of HIV-Infected Malnourished Children in Sub-Saharan Africa AIDS Research and Treatment 2012 2012 790786 22606378 10.1155/2012/790786 PMC3353143

[B40] Manary MJ Sandige HL Management of acute moderate and severe childhood malnutrition BMJ 2008 337 1227 1230 10.1136/bmj.a1227 19008271

[B41] Trehan I Goldbach HS LaGrone LN Meuli GJ Wang RJ Maleta KM Manary MJ Antibiotics as part of the management of severe acute malnutrition N Engl J Med 2013 368 425 435 10.1056/NEJMoa1202851 23363496 PMC3654668

[B42] WHO Community-based management of severe acute malnutrition 2007 Geneva: World Health Organization, World Food Programme, United Nations System Standing Committee on Nutrition, United Nations Children’s Fund

[B43] Creek KE St Hilaire P Hodam JR A comparison of the uptake, metabolism and biologic effects of retinol delivered to human keratinocytes either free or bound to serum retinol-binding protein J Nutr 1993 123 356 361 8429387 10.1093/jn/123.suppl_2.356

[B44] Celik M Sermatov K Abuhandan M Zeyrek D Kocyigit A Iscan A Oxidative status and DNA damage in chidren with marasmic malnutrition J Clin Lab Anal 2012 26 161 166 10.1002/jcla.21505 22628231 PMC6807322

[B45] Yang H De Onis M Algorithms for converting estimates of child malnutrition based on the NCHS reference into estimates based on the WHO Child Growth Standards BMC Pediatr 2008 8 19 10.1186/1471-2431-8-19 18457590 PMC2390546

