# Supplementary material for: Review of the safety and efficacy of vitamin A supplementation in the treatment of children with severe acute malnutrition
Source: Nutr J. 2013 Sep 12;12:125. doi: 10.1186/1475-2891-12-125 (PMC3850897; doi:10.1186/1475-2891-12-125)
Supplement: Additional file 1: Table S1 — Observational studies included in systematic review [45]. [file 1475-2891-12-125-S1.doc]

Additional 1 **Observational studies included in systematic review**

| **Quality Assessment** | | | | | | **Summary of Findings** | | | | **GRADE Rating** |
| --- | --- | --- | --- | --- | --- | --- | --- | --- | --- | --- |
|  |  |  |  |  |  | **Treatment Groups (sample size)** | | **Effect** | |  |
| **Ref** | **Design** | **Indirectness** | **Inconsistency** | **Imprecision** | **Study Limitations (risk of bias)** | **Intervention Cases** | **Control Comparison** | **Relative Risk (95% CI)** | **Absolute** |  |
| de Fátima Costa Caminha,2008 [15] | Observational | No VA intervention | Nutrition def: “normal” WHZ > -1 (NCHS references [45]) | Small sample | Selection bias: age and maternal schooling higher in normal group | PEM (n = 34) | Normal (n = 29) |  |  | LOW-MOD |
|  | Aim: assess and compare serum retinol concentrations in hospitalized Brazilian children (< 60 mo) with protein-energy malnutrition (PEM) | Pop: hospitalized children | VAD def: low serum retinol (< 0.70 μmol/L) |  | Confounding: after adjusting for gender, age, maternal schooling, family income, breastfeeding practices and residence, serum retinol similar in PEM and normal | Prevalence of low serum retinol 41.2% | 24.1% |  | NS (after adjustment |  |
| Ashour, 1999 [14] | Observational | No VA intervention | No serious inconsistency | Small sample; sub-group analyses only possible for PEM arm | Selection bias: inclusion criteria unclear | Hospitalized (n = 68) | Healthy children from community (n = 22) |  |  | LOW-MOD |
|  | Aim: evaluate antioxidant status of hospitalized Egyptian children (3 mo - 3 yr) with PEM | Pop: 68% (46/68) hospitalized children with PEM; 38% with kwashiorkor (KWO) and 29% with marasmus (MAR) |  |  | Controls matched on age and sex; no SES characteristics reported | KWO/MAR plasma A↓ mean glutathione peroxidase ↓ plasma E↓ plasma C ↓ ceruloplasmin↓ copper↓ selenium↓ erythrocyte SOD ↑ plasma Zn↑ |  |  | p < 0.05 |  |
|  |  |  |  |  | Baseline nutrition: HAZ, WAZ, WHZ, BMI significantly different | KWO Hb ↓plasma free Fe↑ |  |  | p < 0.05 |  |
| Mitra, 1998 [18] | Observational | No VA intervention | Nutrition def: NCHS references | Statistics: independent association of WAZ and infection (*S.* *dysenteriae*) with low serum retinol concentration demonstrated in regression modeling | Potential confounding: children with *S. dysenteriae* had higher body temperature, CRP, and AGP concentrations | *S. dysenteriae* (n = 49) Other *Shigella* (n = 14) | Other dysentery (n = 24) |  | p < 0.0001 | LOW-MOD |
|  | Aim: examine relation of serum retinol in Bangladeshi children (5 mo - 5 y) with dysentery due to shigellosis and other nutrition and illness severity indicators | Pop: only children with shigellosis; prevalence of PEM not reported, although mean WAZ < -2 in all groups | VAD def: serum retinol concentration; deficient < 0.35 µmol/L |  |  | Low serum retinol: *S. dysenteriae* CRP low WAZ |  |  | p < 0.0001 |  |
|  |  |  |  |  |  | Serum retinol concentrations higher at discharge (0.36 ± 0.22) than at baseline (1.15 ± 0.50) without supplementation (VAS) |  |  | p < 0.0001 |  |
| Donnen, 1996 [16] | Observational | No VA intervention | VAD def: serum retinol concentration; deficient < 0.35 µmol/L; relative dose response (RDR) | Statistics: no regression modeling | No control or comparison group used | Total sample (n = 415) | No comparison group |  |  | LOW-MOD |
|  | Aim: assess VA status among Zairian children (< 6 yrs | Pop: all children < 6 yrs in 2 villages; mean WAZ -2.24; mean HAZ -2.63; mean WHZ -0.87 | Nutrition def: NCHS references |  | Confounding: no adjustments for possible confounding factors, in particular age | VAD: night blindness 0.7% low serum retinol 19.7% RDR in reduced sample 7.6% |  |  |  |  |
|  |  |  |  |  | Other biases: intrahousehold clustering not accounted for | Low serum retinol: WAZ, HAZ, MUAC |  |  | NS |  |
|  |  |  |  |  |  | Low serum retinol: WHZ < -2 |  |  | p < 0.05 |  |
| Mahalan-abi­s, 1991 [17] | Observational | No VA intervention | VAD def: xerophthalmia – Bitot’s spots, corneal lesions, night blindness, or night blindness plus conjunctival xerosis | No serious imprecision | Selection bias: surveillance data used; urban poor households likely with care-seeking behavior | Cases: xerophthalmia (n = 66) | Control (n = 2,621) |  |  | LOW-MOD |
|  | Aim: determine effect of breastfeeding on risk of xerophthalmia in Bangladeshi children (6 - 35 mo) | Pop: children entered in surveillance system from 1983-1985 aged 6-35 mo; prevalence of PEM not reported | Nutrition def: severe PEM (< 60% reference median WAZ); NCHS references |  | Confounding: many covariates considered | Breastfeeding |  | AOR 0.26 (0.14 - 0.49) | p < 0.001 |  |
|  |  |  |  |  |  | Severe PEM |  | AOR 3.8 (1.8 - 8.0) | p < 0.001 |  |
|  |  |  |  |  |  | Moderate malnutrition (60-70% WAZ) |  | AOR 0.72 (0.28 - 1.8) | NS |  |
|  |  |  |  |  |  | Prolonged diarrhea (10 - 14 days) |  | AOR 5.3 (2.2 - 12.5) | p < 0.001 |  |
|  |  |  |  |  |  | Persistent diarrhea (>14 days) |  | AOR 3.5 (1.8 - 6.8) | p < 0.001 |  |
|  |  |  |  |  |  | Other significant predictors: recent measles and poor SES |  |  |  |  |

The table presents the included observational studies in the review by date of publication, from most recent to oldest. For each study, the design, aim, quality assessment by GRADE criteria, findings, and GRADE rating are provided.
